# Concurrent Peritonsillar Abscess and Uvular Hydrops in a Pediatric Patient

**DOI:** 10.7759/cureus.21701

**Published:** 2022-01-28

**Authors:** Rissa A Zudekoff, Maria F Pugliese, Merlin C Lowe

**Affiliations:** 1 Pediatrics, University of Arizona College of Medicine - Tucson, Tucson, USA; 2 Pediatrics, Banner-Diamond Children's Medical Center, Tucson, USA

**Keywords:** oropharynx, haemophilus influenzae, oropharyngeal infections, peritonsillar abscess, uvular hydrops

## Abstract

Although peritonsillar abscesses (PTAs) are a common presentation in pediatric patients, there are very few reports on a pediatric patient with both a PTA and uvular hydrops. Our patient presented to the emergency room after being unsuccessfully treated for pharyngitis, with odynophagia, dyspnea, muffled voice, drooling, and trismus. On physical examination, we observed a PTA as well as an edematous and erythematous uvula. Following the standard of care, the patient underwent a needle aspiration in the emergency department and subsequently was admitted overnight for observation. The patient had great symptom relief after undergoing drainage of his PTA and was discharged the next morning with symptom resolution of his dyspnea and odynophagia. We recommend drainage and close monitoring for airway compromise as an appropriate treatment course for PTAs and concurrent uvular hydrops.

## Introduction

Peritonsillar abscesses (PTAs) are relatively common in pediatric patients and make up the most common deep infection of the head and neck [1]. Patients with a PTA can present with throat pain, difficulty swallowing, malaise, and often fever. Patients often speak with a muffled voice and present with tender cervical lymphadenitis on the affected side. When examining the patient's oropharynx, erythema and edema of the infected tonsillar pillar and soft palate is usually noted with contralateral deviation of the uvula. It is important to quickly recognize and treat PTAs to avoid further complications consisting of airway obstruction, aspiration pneumonitis or lung abscess secondary to abscess rupture, infection of the deep tissue of the head and neck, hemorrhage secondary to erosion or septic necrosis into carotid sheath, and post-streptococcal sequelae [1]. Uvular hydrops is usually a self-limited idiopathic process and rarely causes as many complications as PTAs. The most common etiologies of uvular edema are allergic reaction or hereditary angioedema with occasional reports related to infectious etiology [2]. Hydrops of the uvula in association with a PTA is a very rare occurrence and has not previously been reported to occur in pediatric patients, to our knowledge. We were able to identify only one prior case report published of a PTA presenting with uvular hydrops, although this was in an adult patient [2]. We present the first reported pediatric case of a 16-year-old boy who had a PTA with associated hydrops of the uvula.

## Case presentation

A 16-year-old boy presented to the emergency department with a left-sided peritonsillar abscess that developed three days earlier. The patient was initially seen in urgent care, where he tested negative for severe acute respiratory syndrome coronavirus 2 (SARS-CoV-2) and Group A streptococcus (GAS) and was sent home with a 10-day course of amoxicillin. Three days later, after only completing two days of the amoxicillin treatment, he re-presented to the emergency room. He endorsed odynophagia, severe pharyngitis, and trismus. He also displayed signs of a muffled voice and mild drooling due to pain associated with deglutition. The patient’s pain radiated to his jaw and behind his left ear, but he denied difficulty in breathing.

**Figure 1:**
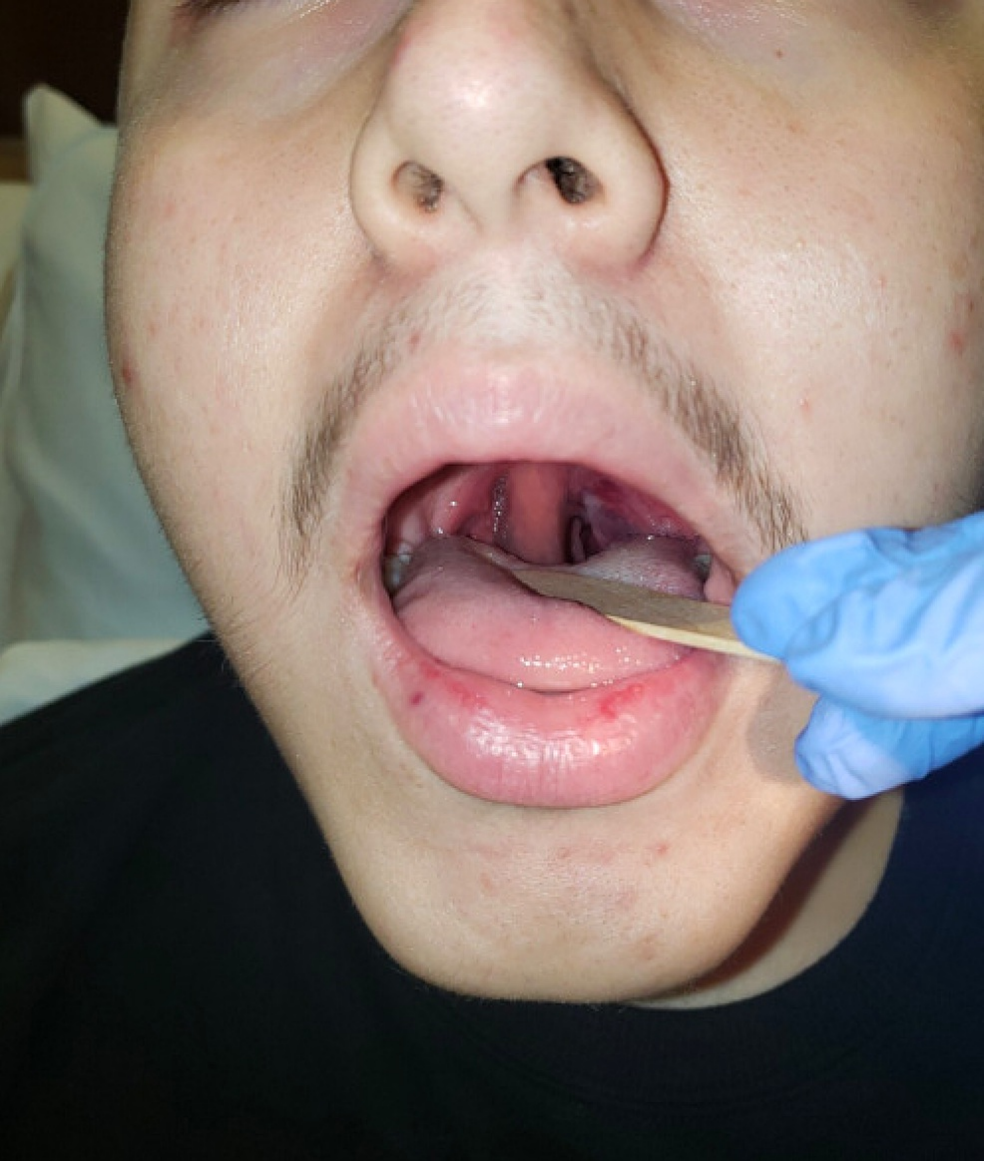
Left peritonsillar abscess and hydrops of the uvula

Physical exam revealed an edematous and erythematous uvula (Figure 1), with the base unable to be visualized, as well as an erythematous, left peritonsillar abscess extending to the roof of the oral cavity and an erythematous soft palate. There was no drainage from the site. Bilateral cervical lymphadenopathy was noted. The patient denied sick contacts, fever, nausea/vomiting, or respiratory distress.

In the emergency department, a needle aspiration of the PTA was performed and 8cc of the purulent fluid from the left peritonsillar abscess was collected. No cultures were sent. Nasal laryngoscopy was performed to rule out epiglottis, which can lead to airway obstruction and respiratory failure. With this view, the uvula was noted to extend into the hypopharynx just above a normal-appearing, unobstructed epiglottis. He was given one dose of Decadron to help with airway edema. He was also started on IV ampicillin/sulbactam 3g every six hours for the treatment of the peritonsillar abscess and IV fluids due to inability to tolerate oral intake. He was observed overnight for monitoring for airway compromise, by pulse oximetry and serial exams, and inability to tolerate oral intake.

## Discussion

Peritonsillar abscesses are common deep neck infections in children. The incidence is approximately 1 in 6000 to 10,000 per year [3]. They occur when there is a collection of purulent fluid between the muscle of the pharynx and palatine tonsils. Infections of the peritonsillar region are usually preceded by pharyngitis or tonsillitis. PTAs are often polymicrobial, but the predominant species found is GAS [4]. The initial evaluation when suspecting PTA is airway assessment, which is based on clinical signs including the ability to speak, respiratory rate, pulse oximetry, and presence of inspiratory stridor. Clinical presentation of PTAs may include unilateral sore throat, trismus, fever, malaise, muffled voice, and a swollen tonsil with uvular deviation. There is usually more pain in the throat on the affected side and referred pain to the ear may also be present. Drooling may develop due to intense pain with swallowing. On examination, the oropharynx typically shows inferiorly displaced tonsils along with erythema and edema of the soft palate.

Complications of PTAs include airway obstruction, aspiration pneumonitis, an extension of the infection to deep neck structures or to the mediastinum, and possible death from secondary hemorrhages or septic necrosis. If the causative organism was GAS, rheumatic fever or post-streptococcal glomerulonephritis may develop. PTA is a clinical diagnosis, and the differential includes peritonsillar cellulitis, retropharyngeal abscess, mononucleosis, or lymphoma. Treatment for PTAs typically consists of surgical drainage through needle aspiration, incision and drainage, or tonsillectomy [1]. Needle aspiration is the preferred treatment in addition to a course of antibiotics [1]. Empiric therapy typically consists of either oral or intravenous ampicillin/sulbactam or clindamycin. Over 90% of patients with drainage of the PTA along with antibiotic therapy will result in PTA resolution [3]. There have been contradictory reports on the use of corticosteroids in the treatment of PTAs. A few studies have shown that corticosteroids decrease edema, improve pain, and decrease recovery time, while other studies show no clear evidence [5,6].

Hydrops of the uvula is rare, usually idiopathic, and infrequently causes serious injury. There have been several reports of uvular hydrops related to an infectious etiology; *Haemophilus influenzae*, GAS, and *Streptococcus pneumoniae* have known associations [7]. This is also a clinical diagnosis and may be associated with pharyngitis, odynophagia, or dysphagia. The uvula may be visualized as erythematous or pale with different severities of swelling. The pharyngeal region may also be erythematous or have exudates. Most patients with this condition are treated symptomatically; few require antibiotics. PTAs and uvular hydrops are typically managed outpatient. To our knowledge, PTA in association with uvular hydrops has not previously been reported in a pediatric patient and, accordingly, little is known about its management and illness course. Our patient did well with treatment of his PTA with observation in the hospital overnight.

## Conclusions

Peritonsillar abscess and uvular hydrops independently have a low risk of causing severe airway compromise, but there is little data to support the outcome when both conditions are observed together. Patients with concurrent conditions should be monitored for complications. Our patient had symptomatic relief after needle aspiration and the laryngoscopy reassured that the epiglottis was unobstructed, allowing the patient to be observed overnight and sent home the following morning. We suggest that drainage and management of the PTA along with close monitoring for airway compromise may be an appropriate treatment course for PTA and concurrent uvular hydrops.
